# Oscillatory EEG Correlates of Arithmetic Strategies: A Training Study

**DOI:** 10.3389/fpsyg.2012.00428

**Published:** 2012-10-19

**Authors:** Roland H. Grabner, Bert De Smedt

**Affiliations:** ^1^Institute for Behavioral Sciences, Swiss Federal Institute of TechnologyZurich, Switzerland; ^2^Faculty of Psychology and Educational Sciences, University of LeuvenLeuven, Belgium

**Keywords:** arithmetic, theta oscillations, alpha oscillations, memory and learning, problem solving, strategy training, EEG, memory retrieval

## Abstract

There has been a long tradition of research on mathematics education showing that children and adults use different strategies to solve arithmetic problems. Neurophysiological studies have recently begun to investigate the brain correlates of these strategies. The existing body of data, however, reflect static end points of the learning process and do not provide information on how brain activity changes in response to training or intervention. In this study, we explicitly address this issue by training participants in using fact retrieval strategies. We also investigate whether brain activity related to arithmetic fact learning is domain-specific or whether this generalizes to other learning materials, such as the solution of figural-spatial problems. Twenty adult students were trained on sets of two-digit multiplication problems and figural-spatial problems. After the training, they were presented with the trained and untrained problems while their brain activity was recorded by means of electroencephalography (EEG). In both problem types, the training resulted in accuracies over 90% and significant decreases in solution times. Analyses of the oscillatory EEG data also revealed training effects across both problem types. Specifically, we observed training-related activity increases in the theta band (3–6 Hz) and decreases in the lower alpha band (8–10 Hz), especially over parietooccipital and parietal brain regions. These results provide the first evidence that a short-term fact retrieval training results in significant changes in oscillatory EEG activity. These findings further corroborate the role of the theta band in the retrieval of semantic information from memory and suggest that theta activity is sensitive to fact retrieval not only in mental arithmetic but also in other domains.

## Introduction

While mathematical proficiency represents an important goal that one needs to achieve throughout education, many children experience difficulties in picking up these competencies. A pivotal objective of educational psychological science thus deals with the understanding of the (neuro)cognitive processes that underlie this development of mathematics, which provides us with crucial information to devise appropriate learning environments (Kilpatrick et al., 2001). The present study focused on the development of different strategies that are used when solving arithmetic problems. Specifically, we used electroencephalography (EEG) to examine how the neural processes that underlie arithmetic strategies change as a function of training. In this vein, our study adds to a growing body of data showing that the human brain is remarkably plastic and that its structure and function is shaped through education and intervention (e.g., Kelly and Garavan, 2005; Draganski and May, 2008; Zamarian et al., 2009).

There has been a long tradition of research in the fields of mathematics education and cognitive psychology, which revealed that children and adults use different strategies to solve elementary arithmetic problems, such as 6 + 9 = or 4 × 3 = (e.g., Siegler, 1988; Lefevre et al., 1996; Siegler et al., 1996; Campbell and Xue, 2001; Geary, 2004). These problems are solved either by directly retrieving the correct answer from long-term memory (retrieval strategy), or by using more time-consuming effortful procedural strategies, such as counting or transforming the problem into smaller sub-problems to arrive at the correct solution (e.g., 7 + 8 = 7 + 3 + 5 = 15). Children progress throughout development from initially using mainly effortful procedural strategies, such as finger counting, to an increasing reliance on retrieval strategies (Siegler et al., 1996), yet both strategies continue to exist into adulthood. It is important to note that difficulties in this development constitute the hallmark of children with mathematical difficulties, who experience problems in both the acquisition of procedural strategies and arithmetic facts (e.g., Geary, 2004; Berch and Mazzocco, 2007). Against this background, it is important to understand the cognitive as well as neuronal processes that underlie these different strategies and their development.

Neurophysiological studies have recently begun to investigate brain correlates of arithmetic strategy use (for a review, cf. Zamarian et al., 2009), providing a new level of analysis that goes beyond behavioral data and that adds new insights into the cognitive processes of arithmetic strategy use and its development (cf. De Smedt et al., 2010). In the majority of these studies, strategy use was examined by contrasting the brain responses between problems in which the size of the operands was systematically varied, assuming that different problem sizes would trigger different types of strategies. More specifically, it has been widely established that in skilled adults problems with smaller operands (i.e., sums ≤ 10; small problems) are more frequently solved by means of fact retrieval than problems with larger operands (i.e., sums > 10; large problems), whereas procedural strategies occur more often in large compared to small problems (Lefevre et al., 1996; Campbell and Xue, 2001).

Following this problem size approach, studies with functional magnetic resonance imaging (fMRI) have provided valuable insights into the brain regions that are involved when different strategies are applied. In general, there is increasing evidence that left-hemispheric perisylvian regions, including the angular gyrus, are consistently more active during the solution of small problems, which indicates that these regions support the retrieval of verbally stored arithmetic facts. On the other hand, a bilateral fronto-parietal network covering the intraparietal sulci is typically more engaged during the solution of problems with larger operands, suggesting the involvement of this network in the application of procedural (calculation) strategies (Stanescu-Cosson et al., 2000; Zago et al., 2001; Kong et al., 2005; Grabner et al., 2007a). These findings are complemented by investigations of event-related potentials (ERPs) in the electroencephalogram of the brain’s electrical activity, which inform us about the time-course of brain activity during arithmetic problem solving. Several ERP-studies have shown that problem size modulates the amplitude of late ERP components occurring after about 300–400 ms (e.g., Jost et al., 2004a,b; Nunez-Pena et al., 2006; Ku et al., 2010), with a more pronounced negativity over right temporo-parietal cortices for large compared to small problems.

In addition to analyses of the functional topography using fMRI and the time-course of brain activity based on ERPs, there is growing interest into oscillatory EEG activity during problem solving, specifically into strategy-related changes in brain oscillations (i.e., induced EEG activity). These changes are related to the coupling and uncoupling of functional networks in the brain and, thus, provide incremental insights into how task-related neuronal networks are formed and interact with each other (Neuper and Pfurtscheller, 2001; Klimesch et al., 2005; Bastiaansen and Hagoort, 2006). Moreover, in contrast to ERPs whose analysis requires averaging over 50–100 trials, reliable measures of induced EEG activity can be obtained based on only a few trials, which makes it a promising candidate for the development of electrophysiological markers of strategy use (De Smedt et al., 2009; Grabner and De Smedt, 2011). A well-established method to quantify induced EEG activity is event-related synchronization (ERS) and desynchronization (ERD; for a review, cf. Neuper and Klimesch, 2006). The ERS/ERD method calculates the percentage amount of band power increases (ERS) or decreases (ERD) in a particular frequency band from a pre-stimulus reference interval to an activation interval (for a more detailed description of this method, see Pfurtscheller and Lopes Da Silva, 2005). Studies applying the ERS/ERD methodology have accumulated much evidence suggesting differential functional significance of various frequency bands. Bandpower increases (ERS) in theta activity (about 3–6 Hz) have been associated with memory encoding and retrieval, in general (Klimesch et al., 1997; Burgess and Gruzelier, 2000; Jensen and Tesche, 2002), and with retrieval of lexical-semantic information from long-term memory, in particular (Bastiaansen et al., 2005; Bastiaansen and Hagoort 2006; Grabner et al., 2007b). Bandpower decreases (ERD) in alpha activity (about 8–13 Hz) have been observed to vary with task difficulty in various cognitive demands (Neubauer et al., 2006; Ku et al., 2010) and thus have been interpreted as general index of invested cognitive resources (Pfurtscheller and Lopes Da Silva, 1999b).

In the domain of mental arithmetic, studies investigating oscillatory EEG activity are rare. Earle et al. (1996) reported higher left-hemispheric theta bandpower when participants solved arithmetic problems compared to inserting an arithmetic sign into an equation, and this increase in bandpower was interpreted to reflect fact retrieval during arithmetic problem solving. Harmony et al. (1999) observed task-related bandpower changes in the theta (increases) and in the alpha band (decreases) during a complex arithmetic task. Theta effects were interpreted to indicate sustained attention, whereas the alpha effect was interpreted as memory retrieval. Micheloyannis et al. (2005) found frontal theta power increases and parietal alpha power decreases during multiplication relative to viewing numbers.

To the best of our knowledge, oscillatory EEG activity related to arithmetic strategy use has only been investigated systematically in two studies. De Smedt et al. (2009) presented adults with small (sums ≤ 10) and large (sums > 10) addition and subtraction problems, which were selected such that the small problems had a large probability to be solved by means of fact retrieval and the large problems had a large probability to be solved with procedural strategies. These authors observed that brain oscillations in the theta and alpha bands were modulated by problem size. In the theta band, solving small compared to large problems was accompanied by higher left-hemispheric ERS. Large problems, in contrast, were associated by bilateral alpha ERD. In both bands, the differences were mainly located in parieto-occipital areas, which may cover those task-related parietal brain regions that have also been observed in fMRI studies (i.e., angular gyrus and intraparietal sulcus). In consideration of the functional significance of the theta and alpha bands, the authors concluded that the pronounced left-hemispheric theta ERS in the small problems reflected the retrieval of arithmetic facts from memory, whereas the strong bilateral alpha ERD in large problems indicated the higher cognitive investment in applying arithmetic procedures.

Most recently, Grabner and De Smedt (2011) conducted an EEG study in which the problem size approach was for the first time complemented by and compared with the information from verbal strategy self-reports. Similar to De Smedt et al. (2009), participants were presented with small and large addition and subtraction problems but they now had to indicate after the solution of each problem which strategy they applied (i.e., fact retrieval vs. application of a procedure). The ERS/ERD data revealed a high general convergence of analyses based on problem size and on strategy reports. Small and self-reported retrieval problems were accompanied by higher left-hemispheric theta ERS, whereas large and self-reported procedural problems were associated by higher bilateral ERD in the alpha band. In the theta band and the upper range of the alpha band (i.e., 10–13 Hz), the differences between conditions were again particularly pronounced over parieto-occipital cortices. In a second analysis, the authors directly compared both approaches with each other and found that self-reported strategy use was linked to the EEG data even when problem size was held constant, for example when only large problems were analyzed. Concretely, higher theta ERS for large retrieval compared to large procedural problems was found, which was most strongly pronounced in the left hemisphere over frontal to centroparietal areas. In contrast, problem size did not modulate ERS/ERD within problems that were reported to be solved using the same strategy (e.g., when only problems that were indicated to be solved by means of a procedure were analyzed). These findings have not only corroborated the functional role of the theta band in arithmetic fact retrieval but also provided the first neurophysiological evidence for the validity of strategy self-reports.

Taken together, the aforementioned studies suggest that strategy use in solving arithmetic problems is reflected in task-related changes in oscillatory brain activity (in particular, in ERS/ERD) in the theta and alpha bands. However, this body of evidence remains to be correlational and static in nature, because it is based on comparisons of brain activity between task conditions that are associated with certain strategies based on problem size or self-reports. As a result, these data do not provide information on how brain activity changes in response to training or intervention. Such information is, however, very relevant for educational psychologists, as it has the potential to reveal insights on the impact of mathematics education on the functional organization of the brain. Against this background, we aim to extend the existing EEG studies by focusing on the effect of fact training on oscillatory brain activity.

In the current high-resolution EEG study, we provided participants with an intensive 2-day fact retrieval training prior to the EEG test session. This allowed us to experimentally manipulate the transition from procedural strategies to fact retrieval use. On the day following the training, participants’ brain activity was recorded while they were solving the trained problems as well as the untrained problems (i.e., problems of a similar difficulty level that they did not receive during the training). In addition, after solving each problem they were asked to report the applied strategy (retrieval, procedural, or other). The comparison of brain responses to trained and untrained problems will reveal those brain areas that are modulated by fact training. A similar approach has already been used in fMRI research on mental arithmetic, which provided complementary evidence supporting the functional distinction at the brain level between arithmetic fact retrieval and procedural strategies, which are supported by the angular gyrus and intraparietal sulci, respectively (Delazer et al., 2003; Ischebeck et al., 2006; Grabner et al., 2009b).

**Figure 1:**
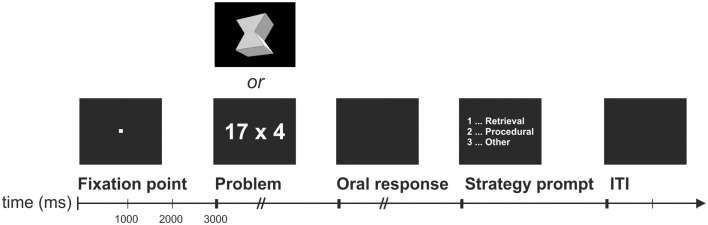
**Schematic display of one EEG trial**. ITI, inter-trial interval of 2000 ms length.

Participants were trained on 10 complex multiplication (two-digit times one-digit) and 10 figural-spatial problems (determination of number of faces in three-dimensional geometric objects; see Figure 1) over 2 days involving a large number of repetitions so that they could easily solve these problems by fact retrieval after completing the training. We selected multiplication problems because these problems are more frequently solved by means of fact retrieval compared to other operations (Campbell and Xue, 2001), and because fMRI training studies have revealed that multiplication facts can be easily acquired even after a short training period – a process that is also reflected in changes in brain activity (Ischebeck et al., 2007). Similar to a previous fMRI training study (Grabner et al., 2009b), we complemented the multiplication problems with a certain type of figural-spatial problems to investigate whether the previously reported changes in theta and alpha activity are specific to arithmetic fact learning or whether they can also be observed in other, non-arithmetical learning tasks in which a particular skill is being automatized. Notably, Grabner et al. (2009b) revealed that the training of both multiplication and figural-spatial problems resulted in similar changes in brain activation, most importantly in activation increases in the angular gyrus.

We hypothesized that the trained problems would be solved faster, more accurately, and more often by (self-reported) retrieval strategies than untrained problems. In line with the presumed functional role of the theta band in fact retrieval, we expected that solving the trained problems would be accompanied by higher theta ERS than solving the untrained problems. This effect was predicted to be particularly pronounced over parieto-occipital regions. Because the untrained problems are more likely to be solved by means of procedural strategies, they should be associated with larger ERD in the alpha band. Similar to Grabner and De Smedt (2011), we distinguished lower and upper alpha frequencies (cf. Klimesch, 1999) and expected a higher topographic differentiation in the upper alpha band. Against the background of the fMRI data reported by Grabner et al. (2009b), we predicted similar training-related changes in theta and alpha band activity for both, the multiplication and the figural-spatial tasks.

## Materials and Methods

### Participants

Twenty-five adult students were recruited through announcements at the universities in Zurich, Switzerland. All participants were healthy, right-handed, and without known mathematical difficulties. Five participants had to be excluded from the analyses (three due to technical problems during EEG acquisition and two because of a lack of valid EEG trials for analyzing retrieval strategy use). The remaining sample of 20 participants consisted of 9 males and 11 females between 21 and 32 years (*M* = 24.30, SD = 2.90). The majority of the participants were psychology students (14/20). The study was approved by the local ethics committee (Swiss Federal Institute of Technology Zurich, Switzerland). All participants gave written informed consent according to the demands of the local ethics committee and were paid for their participation.

### Material

The experimental stimuli comprised 20 multiplication and 20 figural-spatial problems. Similar to previous training studies (Delazer et al., 2003; Ischebeck et al., 2006; Grabner et al., 2009b), the multiplication problems consisted of one operand ranging from 12 to 19 (excluding 15), and one operand ranging from 3 to 8, resulting in products between 42 and 98, excluding solutions divisible by 10. The figural-spatial problems were developed after a figural-spatial subscale of a well-established German intelligence test (Horn, 1983) and were already employed in a previous training study (Grabner et al., 2009b). In each problem, a drawing of a three-dimensional geometric object was presented, requiring the participant to determine the number of object faces as fast and accurately as possible (see Figure 1). Each face is defined by salient angles and can be plane, concave, or convex. For example, a cube consists of six faces whereas a sphere has only one face. This type of figural-spatial problems offers several advantages compared to other problems in this domain: It is a valid measure of figural-spatial abilities (Horn, 1962, 1983; Carroll, 1993), it requires numerical responses similar to the multiplication problems (thus, the two problem types only differ in their cognitive demands but not in their response formats), and the solutions to these problems can be also learned as facts (Grabner et al., 2009b). Initially, 40 figural-spatial problems were created. In a pilot study, we presented these 40 problems together with the 20 multiplication problems to 15 participants. On the basis of these data, we selected 20 figural-spatial problems from the initial set that were matched in task difficulty (in terms of response latencies for correctly solved problems) to the multiplication problems.

In both tasks, half of the problems were included in the training sessions, resulting in 10 trained and 10 untrained multiplication and figural-spatial problems, respectively. The trained and untrained problems were matched with respect to problem size (i.e., numerical magnitude of the solution) and task difficulty.

### Training procedure

Participants underwent a 2-day computer training of 10 multiplication and 10 figural-spatial problems. The training software and a detailed instruction were given to the participants for use on their home computers on a USB stick. Each training session started with a short typing training to familiarize participants with the numerical keypad. In this typing training, random two-digit numbers (40 on the first training day and 20 on the second training day) had to be entered on the numerical keypad as fast as possible. On each training day, the training involved two runs: one run with multiplication and one run with figural-spatial problems (see Grabner et al., 2009b). The order of the runs was randomized. Each run consisted of 15 blocks in which the 10 problems were presented in random order. On the left side of the screen the problem was presented and on the right an empty rectangle appeared into which participants could enter the solution. Participants were instructed to solve the problem as fast and accurately as possible. After giving a solution, positive or negative feedback was provided for 1 s, after which the correct solution was presented for 2 s. The next problem was presented immediately afterwards. To increase training motivation and learning progress, we depicted the number of correctly solved problems and average response latency after each block (i.e., every 10 problems). Before the start of the training on the first day (pre-test) and after completing the training on the second day (post-test), the 10 multiplication and 10 figural-spatial problems were presented once (i.e., one block of multiplication and one block of figural-spatial problems; random order) without feedback in order to quantify the training success. Each training session took about 25–30 min. The training data was automatically saved on the USB stick which was returned at the EEG test session.

### EEG test procedure

The EEG test session took place on the day after the last training. EEG recording started with a 3 min rest EEG during which the participant was instructed to open the eyes, close them, or to deliberately blink, roll, and move the eyes. This sequence was required for the automatic reduction of eye movement artifacts (see below). Next, participants were presented with the 20 trained (10 multiplication and 10 figural-spatial problems) as well as the 20 untrained problems (10 multiplication and 10 figural-spatial problems). Every problem was presented four times, resulting in a total number of 160 trials. The trials were divided into two multiplication and two figural-spatial blocks with 40 items each. Within each block, the trained and untrained problems were presented in random order with the constraint that two consecutive trials could not include the same problem or the same solution. The two multiplication and two figural-spatial blocks were presented in alternating sequence. Nine participants of the final sample started with the multiplication block, the other participants with the figural-spatial block.

The temporal sequence of one EEG trial was similar to Grabner and De Smedt (2011) and is depicted in Figure 1. Each trial started with the presentation of a fixation point for 3000 ms, followed by a multiplication or figural-spatial problem. The participant was instructed to solve the problem as accurately and quickly as possible. In contrast to the computer training, the participants were required to speak the solution into a voice-activated microphone (voice key) that was connected to the recording computer. The time period between problem onset and speech onset (as indicated by the voice key) represented the response latency. The oral response was entered into the computer by the experimenter and also digitally recorded for cross-checking after the test session. A timeout of 7000 ms was applied. Immediately following the response, a strategy prompt appeared in the center of the screen asking participants whether they solved the problem by (a) fact retrieval (e.g., remembering the solution or knowing the solution by heart), (b) application of a procedure (arithmetic: e.g., transformation of the problem or counting; figural-spatial: e.g., counting the number of faces), or (c) any other strategy. They indicated the applied strategy by button press after which a blank screen for 2000 ms was presented as inter-trial interval. At the beginning of the EEG test session, participants were carefully instructed on how to report their strategy use following the procedure described by Campbell and Xue (2001) in their study of arithmetic strategy use. A similar instruction was provided for the participants on how to report their strategy use during the figural-spatial task.

### EEG data acquisition and analysis

**Figure 2:**
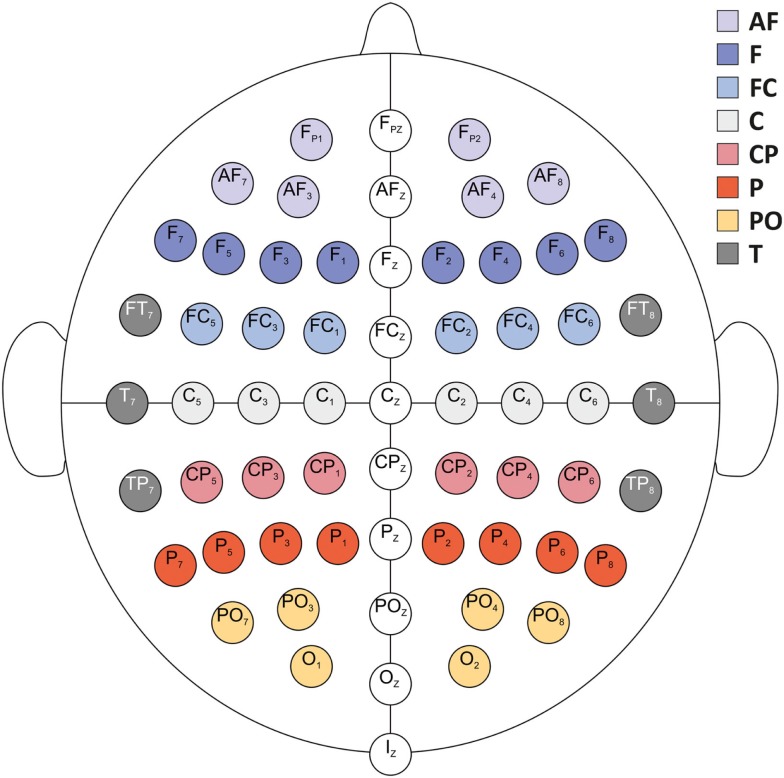
**Schematic display of EEG electrode positions**. For statistical analyses, %ERS/ERD was aggregated over eight areas per hemisphere (electrode positions given exemplarily for the left hemisphere): anteriofrontal (AF; Fp1, AF7, AF3), frontal (F; F7, F5, F3, F1), frontocentral (FC; FC5, FC3, FC1), central (C; C5, C3, C1), centroparietal (CP; CP5, CP3, CP1), parietal (P; P7, P5, P3, P1), parietooccipital (PO; PO7, PO3, O1), and temporal (T; FT7, T7, TP7).

Electroencephalography was acquired through the BioSemi ActiveTwo system (BioSemi, Amsterdam, Netherlands) from 64 scalp electrodes placed according to the extended 10–20 systems (see Figure 2). An electrooculogram (EOG) was recorded from three additional electrodes; two placed horizontally at the outer canthi of both eyes, and one placed above the nasion between in the inner canthi of both eyes. EEG and EOG signals were sampled at 256 Hz and filtered between DC and 128 Hz.

Electroencephalography data analysis was identical to the procedure described in De Smedt et al. (2009) and Grabner and De Smedt (2011). EEG data were first band-pass filtered between 0.5 and 45 Hz to eliminate slow-frequency and power-line contamination artifacts. EOG artifacts were automatically reduced by employing a regression method (Schlogl et al., 2007) based on the resting EEG sequence. The continuous EEG data was then divided into trials of 10 s length (3000 ms before and 7000 ms after problem onset), and all trials were visually inspected for artifacts. The spatial information of the artifact-free EEG data was enhanced by applying a 3D spline surface Laplacian estimation (Babiloni et al., 1998). This procedure has turned out to significantly improve the spatial resolution of EEG potential distributions by reducing head volume conductor effects and by canceling the influence of the electrical reference. ERS/ERD was computed for correctly solved trials only in the theta (3–6 Hz), lower alpha (8–10 Hz), and upper alpha (10–13 Hz) frequency bands (see Klimesch, 1999). To this end, the 10 s trial EEG data was band-pass filtered using a fast Fourier transformation (FFT) based finite impulse response (FIR) method (Oppenheim and Schafer, 1989), yielding a frequency resolution of 0.1 Hz. Afterwards, each amplitude sample of the filtered data was squared by using a moving window (sample-by-sample) of 500 ms length to obtain power values (μV^2^). The data from 500 to 2500 ms after trial onset (during the fixation interval) served as the reference interval (*R*), and the data from problem presentation (at 3000 ms after trial onset) until 125 ms before the oral response as registered by the voice key was used as activation interval (*A*) for ERS/ERD computation. The last 125 ms (i.e., 32 samples) of the response latency were discarded to account for the delay of the voice key trigger signal and to eliminate motor- and speech-related artifacts. For both, *R* and *A* intervals, the bandpower values were first averaged over the respective time intervals (horizontal averaging) and then over the trials (vertical averaging), resulting in two values (one for *R* and one for *A*) per channel. The amount of ERS/ERD was calculated according to the formula: %ERS/ERD = [(*A* − *R*)/*R*] × 100. Positive values indicate increases (ERS) and negative values indicate decreases (ERD) in band power. It is important to note that the length of the activation intervals (*A*) varied between individuals and across trials because these trials were defined as the time period from problem onset until 125 ms before the response. This procedure has been repeatedly used in EEG investigations of higher-order cognitive processes (e.g., Grabner et al., 2004; Neubauer et al., 2004; De Smedt et al., 2009; Grabner and De Smedt, 2011) because the activation interval covers the entire time period of problem solving independently of response latency differences between individuals, task conditions, and trials. For statistical analyses, the %ERS/ERD values were topographically aggregated (by using the arithmetic mean) to obtain eight cortical areas per hemisphere (electrode positions given exemplarily for the left hemisphere): anteriofrontal (AF; Fp1, AF7, AF3), frontal (F; F7, F5, F3, F1), frontocentral (FC; FC5, FC3, FC1), central (C; C5, C3, C1), centroparietal (CP; CP5, CP3, CP1), parietal (P; P7, P5, P3, P1); parietooccipital (PO; PO7, PO3, O1), and temporal (T; FT7, T7, TP7; see Figure 2).

### Statistical analyses

Training data (accuracy, response latency of correct trials) were analyzed using repeated measures ANOVAs including the within-subjects factors Task (multiplication vs. figural-spatial) and Time (pre-test, training session 1, training session 2, post-test). For the behavioral data in the EEG test session, ANOVAs with the within-subjects factors Task and Training (trained vs. untrained problems) were calculated. For EEG data, similar ANOVAs were computed additionally including Hemisphere (left, right) and Area (eight cortical areas as described above) as within-subject factors. ANOVAs on %ERS/ERD data were conducted separately for the three frequency bands (theta, lower alpha, and upper alpha). In all statistical analyses, degrees of freedom were corrected for violations of the sphericity assumption by means of the Huynh–Feldt procedure; the probability of a Type I error was maintained at 0.05. Uncorrected df values together with the corrected *p*-value and the Huynh–Feldt epsilon (ε) were reported when the sphericity assumption was violated. Eta-squared values were calculated as measures of effect size.

## Results

### Training data

**Figure 3:**
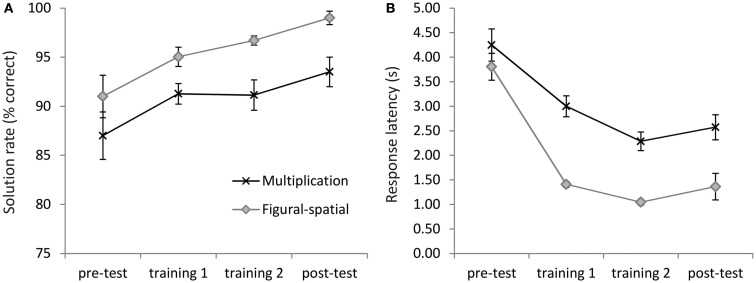
**Training effects on (A) solution rate and (B) response latencies**. Error bars depict 1 SE of the mean.

The 2-day training significantly improved the performance in the multiplication and figural-spatial problems (Figure 3). On average, accuracies increased from 89.00% in the pre-test to 96.25% in the post-test [main effect of Time, *F*(3,57) = 8.13, *p* < 0.01, η^2^ = 0.30, ε = 0.56]. No interaction with Task was observed, suggesting similar increases in accuracy for both types of problems (see Figure 3A). In the response latencies a main effect of Time [*F*(3,57) = 38.21, *p* < 0.001, η^2^ = 0.67, ε = 0.39] emerged, which was additionally moderated by the Task [*F*(3,57) = 5.96, *p* < 0.05, η^2^ = 0.24, ε = 0.44]. As depicted in Figure 3B, the response latencies in the figural-spatial problems revealed a steeper training-related decrease than those in the multiplication problems. There were also main effects of Task for accuracies [*F*(1,19) = 20.56, *p* < 0.001, η^2^ = 0.52] and response latencies [*F*(1,19) = 26.43, *p* < 0.001, η^2^ = 0.58], suggesting that solving the figural-spatial problems was generally easier than solving the multiplication problems (accuracies: 95.43 vs. 90.73%; response latencies: 1.91 vs. 3.03 s; for the figural-spatial and multiplication problems, respectively).

### Behavioral data in the EEG test session

We observed robust and significant effects of Training when comparing trained and untrained problems in both tasks. More specifically, the trained problems were solved more accurately and faster than the untrained problems, as indicated by main effects of Training on both accuracy [93.25 vs. 87.69%; *F*(1,19) = 31.49, *p* < 0.001, η^2^ = 0.62] and response latency [1.47 vs. 2.47 s; *F*(1,19) = 235.67, *p* < 0.001, η^2^ = 0.93]. There were no interactions between Task and Training. Similar to the training data, accuracies were higher (93.94 vs. 87.00%) and response latencies shorter (1.49 vs. 2.45 s) for the figural-spatial compared to the multiplication problems, as indicated by main effects of Task for accuracy [*F*(1,19) = 27.16, *p* < 0.001, η^2^ = 0.59] and response latencies [*F*(1,19) = 37.46, *p* < 0.001, η^2^ = 0.66].

Training also impacted on the self-reported strategy use. Within the correctly solved multiplication problems, 50.42% of the trained compared to 8.57% of the untrained problems were reported to be solved by means of fact retrieval [*t*(19) = 8.16, *p* < 0.001]. Likewise, but even more strongly pronounced, retrieval strategy use was 94.04% and 35.06% on the correctly solved trained and untrained figural-spatial problems, respectively [*t*(19) = 22.16, *p* < 0.001]. Because strategies other than retrieval or procedures were only reported in very few trials (2.96% of the multiplication and 1.68% of the figural-spatial problems), the amount of the procedural strategy use is practically inverse to that of the retrieval strategy use.

### Event-related (de-)synchronization (%ERS/ERD)

**Figure 4:**
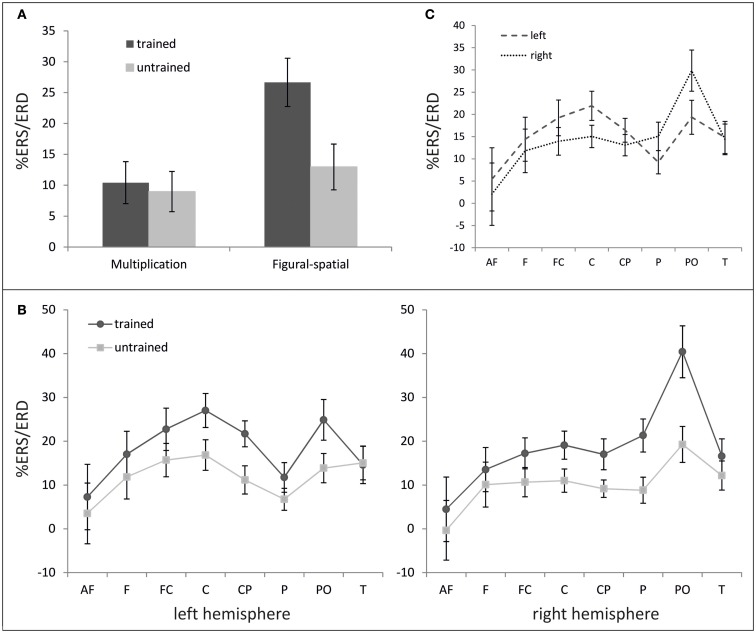
**%ERS/ERD in the theta band (3–6 Hz)**. **(A)** Effect of training as a function of task. **(B)** Topography of training effect. **(C)** Hemispheric differences depending on cortical area. Error bars depict 1 SE of the mean. AF, anteriofrontal; F, frontal; FC, frontocentral; C, central; CP, centroparietal; P, parietal; PO, parietooccipital; T, temporal.

In the theta band (Figure 4), main effects of Training [*F*(1,19) = 19.83, *p* < 0.001, η^2^ = 0.51] and Task [*F*(1,19) = 9.88, *p* < 0.01, η^2^ = 0.34] were observed, suggesting generally higher theta ERS for trained compared to untrained problems (18.53 vs. 10.97%) and figural-spatial compared to multiplication problems (19.81 vs. 9.70%). As depicted in Figure 4A, the training effect was additionally moderated by Task [Training × Task; *F*(1,19) = 14.62, *p* < 0.01, η^2^ = 0.44], indicating that the difference between trained and untrained problems was larger in the figural-spatial than in the multiplication problems. The training effect also interacted with Area [Training × Area; *F*(7,133) = 5.23, *p* < 0.01, η^2^ = 0.22, ε = 0.57] as well as with Area and Hemisphere [Training × Area × Hemisphere; *F*(7,133) = 3.19, *p* < 0.01, η^2^ = 0.14, ε = 0.81]. These interactions are shown in Figure 4B and indicate that the training effect was the strongest over parieto-occipital regions in the right hemisphere. The topographic dominance of the (right) parieto-occipital region was also evident in the main effect of Area [*F*(7,133) = 4.73, *p* < 0.05, η^2^ = 0.20, ε = 0.31] and the interaction between Area and Hemisphere [*F*(7,133) = 4.62, *p* < 0.05, η^2^ = 0.20, ε = 0.35; see Figure 4C].

**Figure 5:**
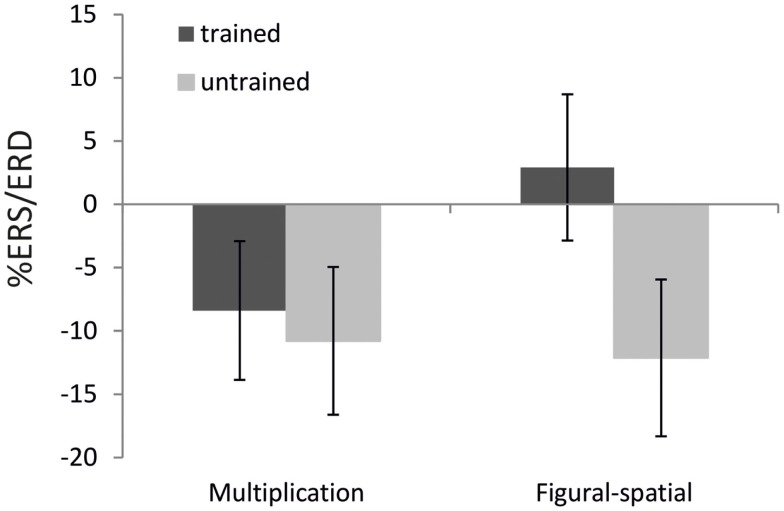
**Effect of training as a function of task on %ERS/ERD in the lower alpha band (8–10 Hz)**. Error bars depict 1 SE of the mean.

**Figure 6:**
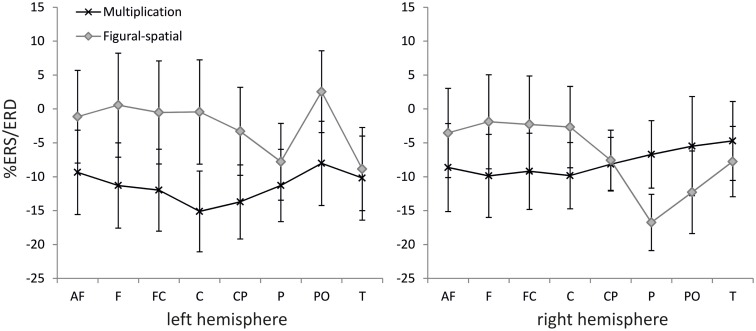
**Effect of task on %ERS/ERD in the lower alpha band (8–10 Hz)**. Error bars depict 1 SE of the mean. AF, anteriofrontal; F, frontal; FC, frontocentral; C, central; CP, centroparietal; P, parietal; PO, parietooccipital; T, temporal.

The ANOVA in the lower alpha band also revealed a main effect of Training [*F*(1,19) = 9.25, *p* < 0.01, η^2^ = 0.33], which was further moderated by Task [*F*(1,19) = 6.67, *p* < 0.05, η^2^ = 0.26]. A higher alpha ERD was found in the untrained compared to the trained problems (−11.46 vs. −2.74%), and the training effect was stronger in the figural-spatial compared to the multiplication problems (see Figure 5). All other effects were related to the Task. The Task × Hemisphere interaction showed that solving the multiplication problems was accompanied by stronger left (compared) to right-hemispheric ERD, whereas the figural-spatial problems were associated with stronger right than left-hemispheric ERD [*F*(1,19) = 13.26, *p* < 0.01, η^2^ = 0.41]. This was also reflected in the interaction between Task, Hemisphere, and Area [*F*(7,133) = 2.65, *p* < 0.05, η^2^ = 0.12, ε = 0.56], which indicated that solving multiplications particularly induced ERD in the left hemisphere whereas the figural-spatial problems were associated with more ERD in the right hemisphere, particularly in the right parietal region (see Figure 6). The interaction between Task and Area was also significant [*F*(7,133) = 4.13, *p* < 0.01, η^2^ = 0.18, ε = 0.46].

In the upper alpha band, there were no significant effects of Training or Task, apart from a marginally significant interaction between Task and Hemisphere [*F*(1,19) = 4.07, *p* = 0.06, η^2^ = 0.18], which revealed a larger hemispheric ERD difference (in favor of the right hemisphere) in the figural-spatial compared to the multiplication problems. Main effects of Area [*F*(7,133) = 4.69, *p* < 0.01, η^2^ = 0.20, ε = 0.48], Hemisphere [*F*(1,19) = 5.58, *p* < 0.05, η^2^ = 0.23], and the Area × Hemisphere interaction [*F*(7,133) = 6.25, *p* < 0.001, η^2^ = 0.25, ε = 0.63] revealed higher alpha ERD in the right compared to left hemisphere. This was especially pronounced over parieto-occipital regions, where also the largest overall alpha ERD was observed.

## Discussion

In the present high-resolution EEG study we investigated how a 2-day training focusing on fact retrieval changed oscillatory brain activity related to different arithmetic strategies. By administering both, an arithmetic (multiplication) and a non-arithmetic (figural-spatial) task, we also examined how changes in EEG theta and alpha activity were related to specific arithmetic demands. Our results reveal strong training effects on performance, self-reported strategy use, and ERS/ERD in the theta and (lower) alpha band across both arithmetic and figural-spatial tasks. The data also indicate that even a short training can induce significant and specific changes in the brain’s electrical activity.

Analysis of the training success and behavioral data in the EEG test session converged to the conclusion that the training worked as expected. Participants became significantly more accurate and faster over the two training days. This was also accompanied by faster and more accurate performance in the trained compared to the untrained problems in the EEG test session. These findings suggest that participants underwent the predicted transition from slower and more effortful procedural to faster and more accurate fact retrieval strategies (Siegler et al., 1996). This is also confirmed by the strategy self-reports that were collected during the EEG test session: Participants indicated that they solved the trained problems more frequently with fact retrieval than the untrained problems. Although the training effect occurred in both tasks, it was more strongly pronounced in the figural-spatial task, as reflected in a steeper training-related decline in response latencies and a higher percentage (more than 90%) of self-reported fact retrieval strategies in the trained problems. This is in line with Grabner et al. (2009b), who administered a similar training over 5 days, and indicates that the figural-spatial facts used in the present study were learned faster than the multiplication facts.

Turning to the oscillatory EEG data, we observed the expected impact of training on theta band activity. More specifically, trained problems were associated with higher theta ERS than untrained problems. This finding extends previous results by De Smedt et al. (2009) as well as Grabner and De Smedt (2011) by showing that theta activity not only correlates with different problem sizes and self-reported strategies but also increases as a function of a short-term strategy training focusing on fact retrieval. Consistent with the behavioral data and the strategy self-reports, the training effect in the theta band was more strongly pronounced in the figural-spatial task compared to the multiplication task. This superior training effect in the figural-spatial task explains why the location of the largest topographic training effect was observed over the right parieto-occipital cortex, as the solution of figural-spatial problems is typically associated with stronger right-hemispheric activation (Vogel et al., 2003).

Thus, our data provide further support for a close association between theta activity and the retrieval of semantic information from long-term memory, which has been proposed for both, language processing (Bastiaansen and Hagoort, 2006) and mathematical cognition (De Smedt et al., 2009; Grabner and De Smedt, 2011). Theta activity is assumed to be involved in functionally linking the cortex with the medial temporal lobe (in particular the hippocampus; Bastiaansen and Hagoort, 2003; Klimesch et al., 2005), which is known to play an important role in memory encoding (Gabrieli, 1998) and in retrieval of facts from memory (e.g., Squire et al., 2004) This assumption fits nicely with findings from several recent fMRI studies, showing that parietal cortical areas as well as the medial temporal lobe are involved in mental arithmetic. For example, there is increasing evidence that the angular gyrus mediates the retrieval of arithmetic facts from memory in adults (e.g., Grabner et al., 2009a; Zamarian et al., 2009) and in children (Rosenberg-Lee et al., 2011). Furthermore, recent data in children suggest the involvement of the medial temporal lobe, in particular the hippocampus, in (early stages of) arithmetic fact learning: De Smedt et al. (2011b) found in 10–12-year-olds higher hippocampal activity in small problems and additions, which are likely solved by means of fact retrieval, compared to large problems and subtraction, which are usually solved by means of procedural strategies by children of this age. Against this background, the observed training-related changes in theta activity may reflect the functional interactions between the medial temporal lobe and parietal cortical structures assumed to support arithmetic fact retrieval processes.

Based on previous findings of stronger alpha ERD in large (compared to small) and self-reported procedural (compared to retrieval) strategies (De Smedt et al., 2009; Grabner and De Smedt, 2011), we also predicted training effects in the alpha frequency range, expecting larger alpha ERD in untrained compared to trained problems. Alpha oscillations are assumed to originate from thalamo-cortical and cortico-cortical networks with their amplitude being inversely related to the activated neuronal population, i.e., larger ERD is associated with larger neural activity (Klimesch et al., 2007). We distinguished between a lower and an upper alpha band (below and above 10 Hz, respectively), since both bands have been repeatedly found to differ in terms of localization of the brain activity (Pfurtscheller and Lopes Da Silva, 1999a, 2005; Neuper and Pfurtscheller, 2001): ERD in the lower alpha band typically emerges widespread over the cortex, whereas ERD in the upper alpha band is topographically restricted to task-relevant areas.

In line with our expectations, the untrained (compared to the trained) problems were associated with larger ERD in the lower alpha band. The untrained problems were solved to a larger extent by more effortful procedural strategies and thus required more cognitive investment compared to the trained problems, whose solutions could be often retrieved from memory. This fits with the functional interpretation of alpha band ERD as a general index of invested cognitive resources (Pfurtscheller and Lopes Da Silva, 1999b). The effect of training was also more strongly pronounced in the figural-spatial than in the multiplication task, similar to the training effects observed in the theta band. This suggests that in general, learning of multiplication problems was more resource-demanding than learning figural-spatial problems.

In contrast to our expectations, no effects of training and task were observed in the upper alpha band. Moreover, the expected task-related topographic differentiation was observed in the lower alpha band with the multiplication problems displaying a pronounced left-hemispheric ERD; the figural-spatial problems, on the other hand, were associated with a strong ERD over right parietal regions. Given the lack of comparable training studies on oscillatory EEG activity in the alpha bands, it is difficult to explain the absence of significant training and task effects in the upper alpha band. However, it is noteworthy that Grabner and De Smedt (2011) also reported a higher sensitivity of the lower alpha band to problem size effects. While problem size strongly modulated lower alpha ERD, no such effects were observed in the upper alpha band. Further studies investigating the functional significance and training sensitivity of different alpha frequency bands are needed.

Similar to the fMRI study by Grabner et al. (2009b), we observed training effects on brain activation in both the multiplication and figural-spatial task. Although there was a general training effect on ERS in the theta band and ERD in the lower alpha band, the effect was stronger in the figural-spatial problems, which resembles the behavioral training data. This finding suggests that the training-related changes in theta and alpha bandpower are not specific to arithmetic but reflect a more general domain-independent mechanism in fact learning. In recent fMRI studies, such a domain-independent mechanism has been proposed for the parietal cortex (specifically, in the angular gyrus; Ansari, 2008; Grabner et al., 2011, in press). These studies suggest that the angular gyrus supports the automatic mapping between overlearned problems and their solutions. In other words, whenever a well-trained problem is presented, the solution to this problem is automatically activated and retrieved from memory. This mapping seems to occur within domains, for example in the connection between an arithmetical problem and its numerical solution, as well as across domains, for example a connection between a figural-spatial problem and a numerical solution (i.e., the number of faces, as in the current training study). Although the present EEG data have a poor spatial resolution and do not allow us to draw firm conclusions on the involvement of specific brain regions in cognitive processes, the observation of the strongest training effects over the parietal and parieto-occipital cortex fits nicely with this account.

The present findings indicate that even a short intervention can induce specific changes in the brain’s activity. Indeed, the human brain is not a static organ but shows remarkable plasticity in response to experiences in the environment (Jäncke, 2009), such as instruction. There is a now growing body of studies in the transdisciplinary field of educational neuroscience that is trying to investigate the effects of different learning environments on brain activity. For example, Rosenberg-Lee et al. (2011) demonstrated that 1 year of schooling had tremendous effects on changes in brain activity during the solution of arithmetical problems in primary school children. Similar effects of instruction on changes in brain structure and activity have been observed in the fields of reading (e.g., Keller and Just, 2009) and video game playing (e.g., Voss et al., 2012). These findings are highly relevant for the field of educational psychology as they add to our understanding of how learning takes place and how it can be fostered. However, it needs to be emphasized that the current sample only included adult participants and that the observed findings may not be readily generalized to children. Future research should therefore aim to replicate the current study in a sample of school-aged children.

The present study also illustrates the value added of brain imaging methods to educational research. Indeed, cognitive neuroscience offers a series of tools and methodologies that allow educational psychologists to complement or extend the knowledge they have already accumulated through decades of behavioral research (e.g., Lieberman et al., 2003; De Smedt et al., 2011a, for a discussion). With the advent of non-invasive brain imaging techniques, it is now possible to investigate at the biological level how people learn. The current findings show that the well-known behavioral shift from effortful procedural strategies to fact retrieval strategies as a function of training is also reflected in specific changes in brain activity. This convergence of findings, obtained by different research methodologies at behavioral and biological levels, provides a more solid empirical ground for our theories on strategy development.

Although the current findings illustrate the effects of training on changes in brain activity, it is important to emphasize that these changes in brain activity may vary as a function of different types of instruction. For example, Delazer et al. (2005) examined the effect of two learning methods, learning by drill (i.e., learn the association between a problem and its answer, as was done in the current study) vs. learning by strategy (i.e., participants learned a sequence of problem solving steps to calculate the solution). They revealed that the drill approach activated the angular gyrus more strongly than the strategy approach. Against this background, future studies should therefore focus more specifically on the effects of different learning environments or instructional approaches on brain activity. Without doubt, such studies will require close collaborations between educational psychologists and cognitive neuroscientists.

## Conflict of Interest Statement

The authors declare that the research was conducted in the absence of any commercial or financial relationships that could be construed as a potential conflict of interest.
